# Editorial: Recent advances in novel therapeutic molecules and targets for inflammatory diseases

**DOI:** 10.3389/fphar.2022.1121821

**Published:** 2023-01-04

**Authors:** Feng Li, Shaogui Wang, Xiaojuan Chao, Shuai Wang

**Affiliations:** ^1^ Infectious Diseases Institute, Guangzhou Eighth People’s Hospital, Guangzhou Medical University, Guangzhou, China; ^2^ Guangdong Provincial Key Laboratory of Translational Cancer Research of Chinese Medicines, Joint International Research Laboratory of Translational Cancer Research of Chinese Medicines, International Institute for Translational Chinese Medicine, School of Pharmaceutical Sciences, Guangzhou University of Chinese Medicine, Guangzhou, China; ^3^ Institute of Precision Medicine, The First Affiliated Hospital, Sun Yat-sen University, Guangzhou, China; ^4^ Institute of Molecular Rhythm and Metabolism, School of Pharmaceutical Sciences, Guangzhou University of Chinese Medicine, Guangzhou, China

**Keywords:** inflammatory diseases, biomarkers, targets, inflammation, compounds

Inflammatory diseases are composed of numerous disorders and conditions that are characterized by inflammation, such as inflammatory bowel disease, hepatitis, and rheumatoid arthritis (Okin et al., 2012). In pathological conditions with inflammatory diseases, the immune system mistakenly attacks healthy cells or tissues, resulting in chronic pain, redness, swelling, stiffness, and damage to the body (Marchetti et al., 2005). Inflammatory diseases have been linked to several potential causes, including diet, stress, and sleep disorders. Anti-inflammatory drugs help to prevent or minimize disease progression. However, common medications used are frequently accompanied by serious adverse effects. There is an urgent need to develop new therapies for inflammatory diseases and elucidate the critical genes and inner mechanism.

Diagnostic biomarkers are useful in the therapeutics of diseases at multiple aspects along the patient’s diagnostic and treatment course. There are various inflammatory biomarkers including cytokines/chemokines, acute phase proteins, immune-related effectors, reactive oxygen and nitrogen species, prostaglandins and cyclooxygenase-related factors, transcription factors, and growth factors (Brenner et al., 2014). Lin et al. demonstrated that lncRNA DLEU2 in the intestinal mucosa was dysregulated upon gut inflammation and could act as a diagnostic biomarker for ulcerative colitis (Lin et al.). They identified DLEU2 as an anti-inflammatory lncRNA which inhibits gut inflammation by negatively regulating the NF-κB signaling pathway (Lin et al.). Huang et al. reported that MHR (monocyte to high-density lipoprotein ratio) and MAR (monocyte to apolipoprotein A1 ratio) were ideal pro-inflammatory markers to reflect bone homeostasis imbalance caused by chronic inflammation in the bone microenvironment of postmenopausal women with type 2 diabetes mellitus (Huang et al.). These researchers expanded the Research Topic of biomarkers to inflammatory diseases.

Finding the right therapeutic targets is the most important approach in anti-inflammatory drug discovery. Many targets are responsible for the anti-inflammatory actions, such as inhibition of cytokine signaling, decreasing leucocyte activation, chemotaxis, and recruitment. Researchers have characterized several targets in this Research Topic. K-Ras is a well-studied oncogene. Qi et al. reported that inhibition of K-Ras^G13D^ mutation promoted cancer stemness and inflammation *via* RAS/ERK pathway (Qi et al.). This finding may be important for understanding the effects of K-Ras^G13D^ mutation on promoting cancer stemness and inflammation when employing targeted therapies to K-Ras^G13D^ mutations in clinical practice. Promyelocytic leukemia zinc finger protein (PLZF) is a transcription factor that acts in regulating a variety of biological processes, such as spermatogenesis, stem cell maintenance, immune regulation, and invariant natural killer T cell (iNKT) development. Hu et al. demonstrated that PLZF, upregulated in mice with non-alcoholic fatty liver disease, was an essential regulator of hepatic lipid and glucose metabolism (Hu et al.). PLZF activates SREBP-1c gene transcription by binding directly to the promoter, inducing repressor-to-activator conversion.

Target-based small molecule drug discovery is an important research direction for inflammatory diseases. Schepetkin et al. designed, synthesized, and evaluated several novel analogs of O-substituted tryptanthrin oxime derivatives as c-Jun N-terminal kinase (JNK) inhibitors. Some of these compounds had a high affinity for JNK1-3 and potently inhibited LPS-induced nuclear NF-κB/AP-1 activation and IL-6 production in human monocytic cells (Schepetkin et al.). Ginsenoside Rc (Rc) is a major component of Panax ginseng. Tang et al. reported that Rc potentially ameliorated inflammatory response and barrier function to protect the gut from DSS-induced colitis (Tang et al.). Zhong et al. showed that Rc alleviated acetaminophen-induced hepatotoxicity by relieving inflammation and oxidative stress (Zhong et al.). Both studies indicated that Rc could be a ligand of FXR by binding with the protein domain. Thus, signaling inhibitors and ligands of nuclear receptors may represent novel anti-inflammatory therapies for the inhibition of multiple cytokines and inflammatory signaling pathways.

**FIGURE 1:**
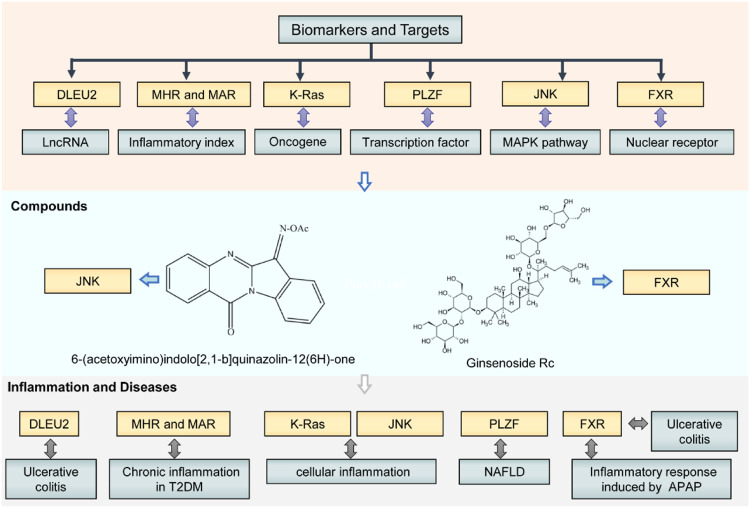
Biomarkers, targets, and compounds for the management of inflammatory diseases in this Research Topic. These biomarkers and targets consist of lncRNA, oncogene, key protein of signaling pathways, and transcriptional factor (e.g., nuclear receptor). Therapeutic agents include natural (e.g., Ginsenoside Rc) and synthetic compounds (e.g., O-substituted tryptanthrin oxime derivatives). NAFLD, non-alcoholic fatty liver disease; T2DM, type 2 diabetes mellitus; APAP, acetaminophen.

Overall, we identified a series of attractive diagnostic biomarkers, therapeutic targets, and compounds for the intervention of inflammatory diseases (Figure 1). These biomarkers and targets consist of lncRNA, oncogene, protein of signaling pathways, and transcriptional factor (e.g., nuclear receptor). Therapeutic agents include natural (e.g., Ginsenoside Rc) and synthetic compounds (e.g., O-substituted tryptanthrin oxime derivatives) (Figure 1). The researchers also delineated intricate gene-chemical relationships and molecular mechanisms in inflammatory disease development. All these findings may yield novel anti-inflammatory lead structures with proven efficacy and pharmacokinetics *in vivo* to manage inflammatory diseases in the future. Further studies would identify the patients who would benefit from a particular anti-inflammatory drug action, making treatment for disease state more personalized and effective.
